# Head Loss As an Explanation of the Steal Phenomenon in Microvascular Surgery

**Published:** 2015-10-06

**Authors:** Phillip E. Ross, Frederic W.-B. Deleyiannis

**Affiliations:** ^a^School of Medicine; ^b^Department of Surgery, University of Colorado, Aurora

**Keywords:** lower extremity, perforator flap, posterior medial thigh flap, steal phenomenon, head loss

## Abstract

Vascular steal has been cited to help explain end-organ ischemia after microvascular reconstruction. Attempts to clarify a mechanism of vascular steal have been made by modeling blood circulation after a simple electrical circuit, suggesting that the free flap provides a path of least resistance for blood flow and thereby compromises end-organ perfusion. We present a case of a posterior medial thigh perforator flap for the reconstruction of a diabetic foot ulcer in a patient with a single vessel providing inflow to the foot. In the context of this case, we provide a novel explanation for the steal phenomenon using the Hagen-Poiseuille law and the property of head loss in fluid dynamics and discuss how the vessel size of the free flap may contribute to a steal phenomenon.

We present a case of a posterior medial thigh perforator flap for the reconstruction of a diabetic foot ulcer in a patient with a single vessel providing inflow to the foot. In the context of this case, we discuss how head loss (ie, decrease in fluid pressure) and the vessel size of the free flap may contribute to a steal phenomenon.

## CASE

**Figure 1:**
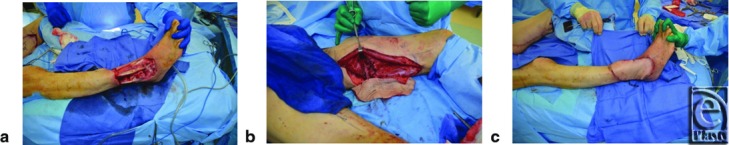
Defect, flap harvest, and flap inset. (a) Lower extremity defect: preoperative vascular workup revealed an occlusion of the anterior tibial artery at its origin, a toe-brachial index of 0.10, and flow to the foot only from the posterior tibial artery. (b) Medial thigh posterior free flap based on a single perforator identified with a Doppler probe and found to traverse the semimembranosus muscle and to originate from the profundus femoral artery. Pedicle length was 6 cm with an artery of 1.0 mm in diameter. (c) Closure, end-to-side anastomosis done to the posterior tibial artery.

An 81-year-old man presented with a 17 cm × 8 cm defect of the right lower extremity after debridement of a diabetic ulcer (Fig. 1*a*). A posterior medial thigh perforator flap was designed by identifying and centering a dominant skin perforator under the skin paddle (Fig. 1*b*). The patient was discharged with well-perfused free flap and foot on postoperative day 14 (Fig. 1*c*). However, over the next 3 months, the patient presented with slowly progressive tissue loss of the distal free flap and dorsal foot skin with the eventual need for an amputation.

## DISCUSSION

**Figure 2:**
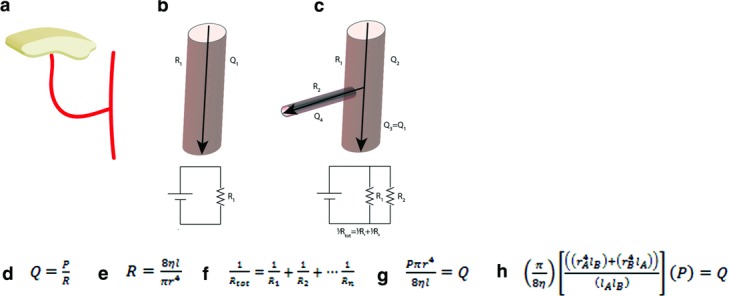
Schematic of vessel anastomosis and flow calculations. (a) A cartoon schematic of the flap and corresponding anastomosis with the main end-organ artery. (b) Schematic of a section of the blood vessel with an arrow showing direction of the flow of blood. *R*_1_ and *Q*_1_ on either side designate the resistance and flow rate, respectively. Below is a schematic of the conduit's analogous electrical circuit with resistor R_1_ shown for clarity. (c) The same section of blood vessel as in (b) is shown with new anastomosis sutured in an end-to-side “T-branch” fashion. Arrows show direction of blood flow. *Q*_2_ represents the flow rate proximal to the junction, which also represents the new overall flow rate. *Q*_3_ = *Q*_1_ represents the flow rate distal to the junction being equal to the flow rate in (b). The electrical circuit shown below is used to help illustrate the addition of the vasculature in parallel, represented by resistors R_1_ and R_2_. *R*_2_ and *Q*_4_ designate the resistance and flow rate of the perforator for the free flap, respectively. (d) The Hagen-Poiseuille law of fluid dynamics, where *Q* is the flow rate, *R* is the total resistance, and *P* is the total pressure at any given location. (e) Resistance experienced by blood traveling in a vessel, where η is the blood viscosity (0.0035 Pa·s), *l* is the vessel length, and *r* is the radius of the vessel. (f) Total resistance for *n* resistors in parallel. (g) Expression for flow rate using the Hagen-Poiseuille law for a single vessel. (h) Expression for flow rate using the Hagen-Posiseuille law when vessels are added in parallel. *A* and *B* designate the radius and length of the main branch and the perforating vessel from the donor flap, respectively.

A concern for microvascular surgeons is adequate perfusion to the lower extremity distal to the recipient site of free flap transfer, especially in the extremity being perfused by a single vessel. This loss of perfusion (ie, “steal phenomenon”) has been attributed to the newly sutured vasculature siphoning blood flow away from the artery providing distal perfusion.^1^ To determine the effect of addition of a new vessel added in parallel, previous authors have cited the Hagen-Poiseuille law of fluid dynamics, which models laminar flow in noncompliant vessels. In addition, principles of resistance in electrical circuitry have been used to explain resistance experienced by fluid flow in vessels^1^^,^^2^ (Fig. 2).

As an example, consider the case reported earlier. Using a diameter of 3 mm for the main vessel (ie, posterior tibial artery) and a length of 100 mm, a flow rate (*Q*_1_) of 5.68*P* (mm^3^/Pa·s) is generated for a given pressure using the equation in Figure 2*g*. To assess the flow through each vessel when the new vessel (ie, perforator for the free flap with a vessel diameter of 1.0 mm and length of 60 mm) is added in parallel, we must consider 3 sections of flow: the flow in the portion of the main artery proximal to the anastomosis (ie, overall flow through the conduit [*Q*_2_]); the flow in the portion of the same artery distal to the anastomosis (*Q*_3_); and the flow in the perforator to the free flap (*Q*_4_). Using the equation given in Figure 2*h*, an overall flow rate (*Q*_2_) of 5.80*P* (mm^3^/Pa·s) is generated for the entire system/conduit when a vessel (ie, the free flap) is added in parallel. To calculate the flow to the free flap, we again use the expression in Figure 2*g* to find that a flow rate of 0.12*P* (mm^3^/Pa·s) is achieved. To adhere to the law of conservation of a liquid, the sum of volumetric flow rate in each vessel must equal the total flow through the conduit.^3^ Therefore, the difference in flow between the artery proximal to the anastomosis (*Q*_2_) and the perforating artery to the free flap (*Q*_4_) is equal to 5.68*P* (5.80*P* − 0.12*P*), which is the flow distal to the anastomosis (*Q*_3_). Thus, as calculated using the Hagen-Poiseuille law, there is no loss of flow through the original main vessel due to the addition of vessels in parallel if pressure remains constant (ie, *Q*_1_ = *Q*_3_).

### HEAD LOSS AS AN EXPLANATION OF THE STEAL PHENOMENON

Simply modeling fluid flow after electrical circuits can be incomplete. A factor that could cause reduction in flow to an end organ could be attributed to head loss. As blood moves through a vessel, some volume of blood will change direction to flow through the new vasculature that has been anastomosed.^3^ This sudden change in direction will change blood flow momentum and therefore reduce the amount of pressure that would otherwise provide blood flow to the end organ.^3^ Head loss is calculated as follows:

**Figure:**
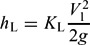


where *h*_L_ is the head loss, and *K*_L_ is the loss coefficient. For perpendicular vessels, values for *K*_L_ range from 0.7 to 0.9.^3^ *V*_1_^2^ is the velocity of blood in the main artery proximal to the anastomosis. *g* is the acceleration due to gravity. Flow rate and velocity can be related by the following expression:

**Figure:**
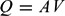


where *A* is the internal area of the vessel, and *V* is the velocity. Because the area of the vessel is not changing, we can therefore directly attribute any change in flow rate to a change in velocity of the fluid. It was found earlier that according to the Hagen-Poiseuille law, flow rate increased (*Q*_2_ > *Q*_1_) and therefore velocity increased when vessels were placed in parallel. This then leads to an increase in head loss.

**Figure 3:**
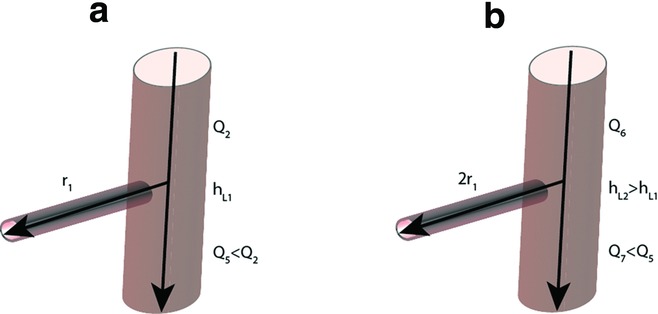
Flow rate schematic. (a) Schematic of blood vessel, where *r*_1_ represents the radius of the vessel perforating the flap, *Q*_2_ represents flow rate proximal to junction, which is the same flow rate in Figure 2*c, h*_L1_ represents the head loss at the junction, and *Q*_5_ < *Q*_2_ represents the flow rate distal to the junction being less than the flow rate proximal to the junction. (b) Schematic of the blood vessel, where 2*r*_1_ represents a doubling of the perforating flap vessel as compared with (a), *h*_L2_ > *h*_L1_ represents greater head loss compared with (a) when the radius of the perforating flap vessel is doubled, and *Q*_7_ < *Q*_5_ denotes the flow rate distal to the junction being less than the flow rate distal to the junction in (a).

Because resistance is inversely proportional to the radius to the fourth power of the vessel, doubling the radius of the flap artery (i.e. the newly anastamosed vessel) will increase the flow rate by a factor of 16 to the flap. This larger pedicle will result in a relatively larger overall flow and velocity (ie, *Q* proximal to the anastomosis increases by 30% when the flap pedicle is doubled in radius (Fig. 2, equations). Because head loss is dependent on the velocity squared, doubling the radius of the perforator to the free flap will increase head loss by 70%, which could result in a larger loss of pressure distal to the anastomosis and a decrease in flow to the end organ (Fig. 3).When selecting the size of an arterial pedicle (ie, diameter) of a free flap, a smaller diameter may lead to less head loss and thus less decreased perfusion (steal) to the foot. In designing a free-style free flap, the proximal dissection to obtain a larger pedicle must be considered as an obvious way not just to obtain a larger vessel to facilitate an easier anastomosis but also to have the potential disadvantage of causing greater head loss and thus vascular steal. In our case, we chose a perforator flap with an arterial diameter of 1 mm to decrease the possibility of vascular steal, but even with this consideration, we feel the perfusion of the foot may have been inevitably compromised. In addition, although the flap appeared circumferentially well-perfused on discharge, the selection of a smaller perforator (vs a larger perforator) may have limited the size of the skin paddle that could be reliably harvested. This may have contributed to the eventual loss of some of the distal skin of the free flap. Thus, in selecting a small perforator for inflow to a free flap, one must also consider the competing advantage and potential disadvantage of less vascular steal to the foot versus the harvest of a smaller free flap. Head loss in the vessel providing inflow to the end organ (ie, foot) may also increase if the microvascular anastomosis introduces turbulent blood flow since greater head loss (ie, resistance) is experienced with turbulent flow with than laminar flow.

While the aforementioned discussion suggests evidence for vascular steal from a fluid dynamics perspective, other factors could also contribute to this phenomenon. Much of the end-organ failure that has been documented has been in patients with diabetes or severe peripheral vascular disease.^1^^,^^4^^-^7 Atherosclerotic vessels tend to have poor response to autoregulatory mechanisms in order to compensate for changes in blood pressure.^8^ Therefore, it is reasonable to conclude that the main perforating artery to the end organ distal to the anastomosis may be unable to compensate the drop in pressure. A more quantifiable method to measure blood flow to the end organ, such as careful measurements with a plethysmograph or more advanced computational methods to model fluid dynamics, is needed to provide additional details for describing the phenomenon of vascular steal.

## Acknowledgments

The authors thank Eric Petersen (School of Medicine, University of Colorado, Aurora, Colo) for his valuable insight and contributions to this work. Without it, this project would not have been possible.
